# The Role of mHealth Interventions in Changing Gender Relations: Systematic Review of Qualitative Findings

**DOI:** 10.2196/32330

**Published:** 2022-07-21

**Authors:** Elizabeth K Kirkwood, Caitlin Clymer, Kheminda Imbulana, Sumaya Mozumder, Michael J Dibley, Neeloy Ashraful Alam

**Affiliations:** 1 Sydney School of Public Health Faculty of Medicine and Health University of Sydney Camperdown Australia; 2 University of Sydney Camperdown Australia

**Keywords:** mobile health, mHealth, gender relations, systematic review, low- and middle-income countries, mobile phone

## Abstract

**Background:**

The rapid and widespread growth of mobile technologies in low- and middle-income countries can offer groundbreaking ways of disseminating public health interventions. However, gender-based inequalities present a challenge for women in accessing mobile technology. Research has shown that mobile health (mHealth) interventions can affect gender relations in both positive and negative ways; however, few mHealth programs use a gender-sensitive lens when designing, implementing, or analyzing programs.

**Objective:**

This systematic review aims to identify and summarize the findings of qualitative research studies that explore the impact of mHealth interventions on gender relations as a result of participating in such initiatives in low- and middle-income countries.

**Methods:**

We performed a systematic literature review to examine empirical evidence of changes in gender relations attributed to participation in an mHealth intervention in low- and middle-income countries. Peer-reviewed articles were included based on whether they evaluated an mHealth intervention and were published between 2013 and 2020. Articles using mHealth that solely targeted health workers, did not assess a specific intervention, used mobile technology for data collection only, or were formative or exploratory in nature were excluded. The search terms were entered into 4 key electronic databases—MEDLINE, EMBASE, PsycINFO, and Scopus—generating a comprehensive list of potentially relevant peer-reviewed articles. Thematic analysis was used to identify, analyze, and report the themes that emerged from our data.

**Results:**

Of the 578 full-text articles retrieved, 14 (2.4%) were eligible for inclusion in the study. None of the articles appraised gender from the outset. The articles uncovered findings on gender relations through the course of the intervention or postprogram evaluation. Most studies took place in sub-Saharan Africa, with the remainder in South and Southeast Asia. The articles focused on maternal and child health, HIV diagnosis and treatment, and reproductive health. This review found that mHealth programs could enhance spousal communication, foster emotional support between couples, improve women’s self-efficacy and autonomy in seeking health information and services, and increase their involvement in health-related decision-making. Despite the positive impacts, some mHealth interventions had an adverse effect, reinforcing the digital divide, upholding men as gatekeepers of information and sole decision-makers, and exacerbating relationship problems.

**Conclusions:**

These results suggest that given the rapid and persistent upscale of mHealth interventions in low- and middle-income settings, it is imperative to design interventions that consider their impact on power dynamics and gender relations. Future research is needed to fill the evidence gaps on gender and mHealth, acknowledging that women are not passive beneficiaries and that they need to actively participate and be empowered by mHealth interventions.

## Introduction

The rapid and widespread growth of mobile technologies, especially in low- and middle-income countries (LMICs), offers an innovative mechanism for disseminating public health interventions [1-3]. The extensive use of mobile devices can reduce the geographical barriers often faced in rural and regional areas, encouraging their inclusion in health care and health-related interventions [1,4]. Mobile phones offer the potential to improve health care by providing accessible, sustainable health care for underserved communities, contending with underresourced health care systems in low- and middle-income settings [5,6]. Over 750 million people, or 10% of the global population, still do not have access to a mobile broadband network [7]. This primarily affects those living in rural and remote areas of LMICs [7]. A further 3.3 billion people who live within the reach of a mobile broadband network do not use mobile internet because of financial barriers, lack of awareness of mobile internet and its potential benefits, and lack of skills or confidence in using mobile internet [7]. Many digital-based health programs aim to improve women’s health in LMICs, often focusing on maternal and child health [8-11]. However, gender-based inequalities pose a challenge for women, who experience lower literacy rates and less access to mobile technology, inhibiting the uptake and impact of health interventions delivered via digital platforms [12-14].

Mobile health (mHealth) is defined by the World Health Organization as any “medical and public health practice supported by mobile devices” [15]. Evidence suggests that mHealth interventions effectively enhance treatment adherence and appointment compliance and can be used as a tool to assist with data collection [2,4]. Research has also shown that mHealth interventions can transform gender relations positively by improving access to health resources, increasing women’s decision-making ability, and supporting spousal communication [12]. mHealth interventions have the potential to increase women’s autonomy in seeking health services and health information, thus enhancing their health-related decision-making [16]. This is because mHealth interventions alter traditional mechanisms for communication with health care professionals and, as such, can reduce or eliminate women’s reliance on spousal approval and financial support to access health services and afford confidentiality and anonymity.

A systematic review by Jennings and Gagliardi [16] revealed the need for a further rigorous investigation into mHealth in terms of implementation and evaluation to establish whether mHealth programs transform rather than reinforce gender inequalities, and this review builds upon these findings [16]. The review highlighted that women face multiple barriers to participating in mHealth interventions, including social, financial, and digital literacy and the need for spousal approval [16]. Research on the effect of mHealth interventions on men’s and women’s interactions highlighted that when scaling up mHealth interventions, it is critical to ensure that the intervention targets the transformation of gender relations and does not reinforce existing gender inequities [16].

The term gender refers to the socially constructed characteristics of women and men and the behavioral norms, relationships, and roles associated with identifying as female or male [17]. Gender relations can be defined as how “a culture or society defines rights, responsibilities, and the identities of men and women in relation to one another” [18]. The relationships between men and women are also influenced by political, economic, religious, environmental, and sociocultural constructs [19]. Therefore, gender significantly affects people’s experiences of and access to health care [17].

It is becoming increasingly evident that mHealth can improve the lives of many; however, there is limited research examining the influence of these interventions on gender-based power dynamics and existing inequalities and their impact on women’s access to health resources [16,20]. However, evidence supports the use of a gender equity lens in designing and analyzing digital programs [20]. In their review of findings from a cohort of implementation research projects in LMICs, Sinha and Schryer-Roy [20] argued that gender and power analyses are essential when designing and implementing digital interventions [20]. Although researchers have noted several positive impacts of mHealth interventions on gender relations, including increased communication between opposite-sex partners, enhanced female autonomy, improved female social status, and increased access to health resources [16], evidence has also suggested that these programs may unintentionally perpetuate the digital divide and enhance pre-existing power imbalances, exacerbating gender inequalities [12,16,21]. Evidence suggests that a lack of gender analysis and health equity when designing, implementing, and evaluating digital interventions can exacerbate or create new health inequity and gender inequalities [20]. However, the absence and low quality of available literature limit analysis on this issue [16]. As the number of mHealth interventions continues to increase, further research is required to illuminate their impact on gender relations, particularly in low- and middle-income settings.

This systematic review aimed to identify and summarize the findings of qualitative research studies that explore the impact of mHealth interventions on gender relations as a result of participating in such initiatives. Are gender relationships adequately assessed when implementing mHealth interventions? This paper examines empirical evidence of changes in interactions between women and men attributed to their participation in an mHealth intervention in an LMIC. In doing so, it aimed to illuminate the risks and benefits of using mHealth interventions in the context of gender relations in LMICs.

## Methods

### Inclusion Criteria

In our review, we included research studies published in peer-reviewed journals that met the following criteria: (1) the study used qualitative research methods to evaluate an mHealth intervention; (2) the study documented findings on the impact of an intervention on gender relations for intervention participants; (3) the study was published in English between January 2013 and December 2020; and (4) the mHealth intervention was conducted in an LMIC, as defined by the 2020 World Bank classification [22].

Studies were excluded if they were conducted in upper- or upper–middle-income countries, published in a language other than English, gray literature, and non–peer-reviewed or unpublished reports (dissertations and conference abstracts). We also excluded publications that did not specifically assess an mHealth intervention, studied mHealth interventions that solely targeted health workers, used mobile technology for data collection only, and were nonintervention studies such as formative research or exploratory studies.

The systematic review is registered with PROSPERO (International Prospective Register of Systematic Reviews; CRD42021218001).

### Search Strategy

The research team conducted a preliminary literature search to identify appropriate search terms relevant to the scope of our review. The electronic search of the Scopus database was the primary means of collating the initial list of appropriate terms. All authors compiled and agreed on relevant search terms and expanded the list to include synonyms and variations in spelling classified under 3 key areas: mHealth, maternal health–related and child health–related terms, and gender relations, as listed in Textbox 1. The key search terms (using Boolean operators) were then entered into 4 key electronic databases—MEDLINE, EMBASE, PsycINFO, and Scopus—generating a comprehensive list of potentially relevant peer-reviewed articles.

**Search strategy for electronic databases.**

**Search category and search terms (searched using Boolean operator AND)**

**Mobile phones**

“Mobile phone(s),” “cell phone(s),” “cellular phone(s),” “mobile,” “phone,” “mobile-based,” “mobile applications,” “SMS,” “text,” “text-message,” “audio message,” “smartphone,” “eHealth,” “mHealth,” and “mobile health”

**Maternal health–related and child health–related interventions**

“Health,” “maternal,” “child,” “birth(s),” “delivery,” “child,” “obstetric,” “pregnancy,” “neonatal,” “antenatal,” “anaemia,” “pre-eclampsia,” “HIV,” “AIDs,” “malaria,” “abortion,” “tuberculosis,” “postpartum,” “family planning,” “sexual,” and “reproductive”

**Gender relations**

“Gender,” “women,” “female,” “relation,” “interaction,” “equity,” “inequity,” “equality,” “inequality,” “men,” “male,” “participation,” “empower,” “sex roles,” “women’s role,” “men’s role,” “gender role,” “autonomy,” “violence,” “gender-based violence,” “intimate partner violence,” “domestic violence,” “safety,” “literacy,” “economic,” “mobility,” “status,” “access,” “capacity,” and “communication”

### Title, Abstract, and Article Screening

**Figure 1:**
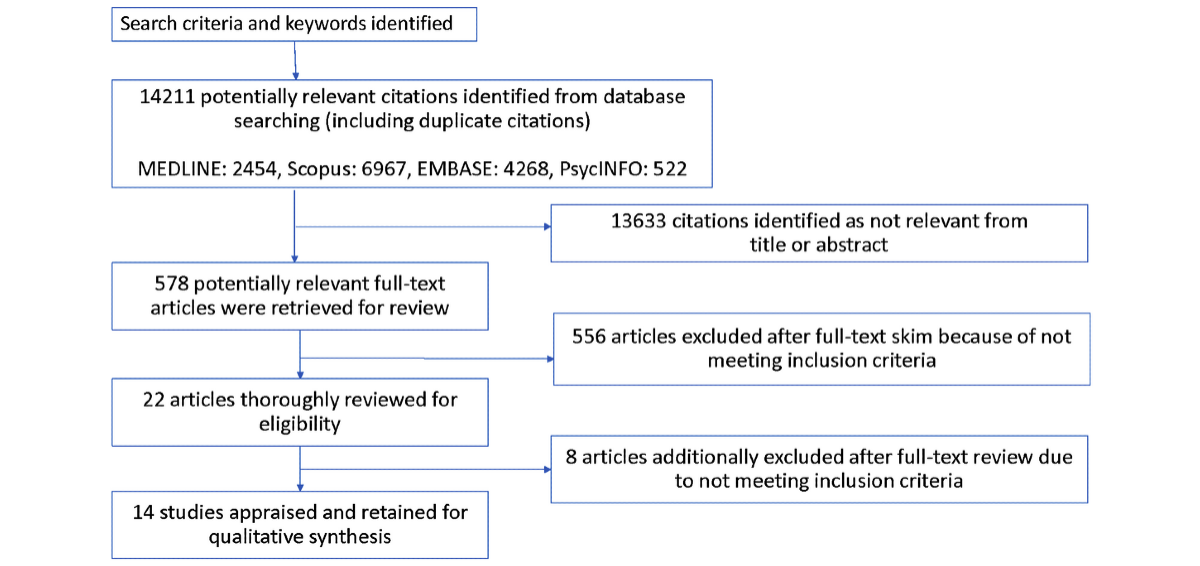
Search screening and flowchart. mHealth: mobile health.

The research team independently reviewed titles and abstracts obtained from the initial results of the electronic databases. The researchers compiled a list of all potentially relevant articles. If the title and abstract did not provide sufficient information, the full-text article was retrieved, saved in Endnote, and assessed for eligibility. Full-text articles were independently skim-read by 4 research team members and included or excluded as per the criteria. The research team shared a Microsoft Excel spreadsheet containing citations and their findings and discussed their results. Any inconsistencies were examined and adjusted based on the consensus of all authors, resulting in a finalized list of publications for review. The search and screening process is outlined in the PRISMA (Preferred Reporting Items for Systematic Reviews and Meta-Analyses) [23] flow diagram in Figure 1.

### Study Appraisal

The final list of the selected studies is shown in the *Characteristics of Included Studies* section. All authors systematically appraised each study, and information was extracted and recorded under the following categories: article number, author, journal, year of publication, description of mHealth intervention, country, study’s primary objective, study design, sample size (qualitative and quantitative), and findings on gender relations.

### Quality Assessment

Each study was independently reviewed by the research team. We assessed qualitative studies using the Critical Appraisal Skills Programme Qualitative Research Studies checklist [24]. Each paper was appraised to grade the quality of evidence using the 10 questions listed in the *Characteristics of Included Studies* section. A score was assigned for each study. The research team debated any discrepancies in scores until all team members agreed to all scores represented. For question 7 regarding ethical considerations, the paper was awarded a score if the research was approved by an institutional ethics committee or review board. Overall, the literature was of high quality and used appropriate methodologies, recruitment strategies, and research designs. All the articles discussed the value of the research and provided a clear statement of the aims and findings. Most of the literature includes appropriate methods for data collection and analysis; however, very few articles discussed reflexivity. The results are presented in the *Characteristics of Included Studies* section.

### Synthesis Process

Each researcher independently reviewed the findings on the influence of mHealth on gender relationships. Data from each publication were coded manually by all 4 researchers, identifying key text that captured the effect of mHealth on gender relations and aligned with our research question. Each researcher read each article several times, made preliminary notes to document and analyze the initial findings, and provided a framework for emerging themes. We reviewed the results using thematic analysis to identify, analyze, and report themes within our data set [25]. The researchers met to share emerging themes to decide how to present the key thematic synthesis findings.

We present our findings based on the framework by Jennings and Gagliardi [16] and report our results under 3 key themes: positive transformative influences, negative transformative influences, and nontransformative influences. Positive transformational influences on gender relations empower women and enhance gender relations. Negative transformational influences disempower or adversely affect relationships. Nontransformative influences perpetuate rather than challenge gender-based disparities [16].

## Results

### Literature Search and Review Process

A total of 14,211 articles were retrieved using our search terms, and the titles and abstracts were reviewed for relevance. Of these 14,211 articles, 578 (4.07%) full-text and peer-reviewed articles were retrieved for review. The articles were skimmed and reviewed for eligibility, with 96.2% (556/578) of articles being excluded because of the absence of an evaluation of an mHealth intervention, the mHealth intervention targeting health workers, lack of reported findings on gender relations, or the study not being conducted in an LMIC. Additional screening of the remaining 22 articles led to a further 8 (36%) articles being excluded because of insufficient information, unclear methodology, or general information regarding mHealth interventions.

### Characteristics of Included Studies

A total of 14 publications were included in the final list of articles [26-39]. Of these 14 studies, 3 (21%) were conducted in Bangladesh, 1 (7%) in Vietnam, and 1 (7%) in India. The remaining interventions were conducted in sub-Saharan Africa, including 29% (4/14) of studies in Kenya, 21% (3/14) of studies in Uganda, 7% (1/14) of studies in Ghana, and 7% (1/14) of studies in Malawi. All selected studies were sourced from electronic databases and found in peer-reviewed journals. The mHealth interventions focused on several health areas, including agriculture and nutrition counseling; maternal, neonatal, and infant health care; sexual and reproductive health; HIV or AIDS and antiretroviral treatment; intimate partner violence (IPV); and health-linked unconditional cash transfers. The mHealth apps used in these studies involved SMS text messages, automated SMS text messages, automated voice messages, access to hotlines and counseling call centers, and interactive voice response (IVR) technology. All studies focused on assessing barriers to and facilitators of mHealth interventions, such as feasibility, acceptability, accessibility, and appropriateness. All studies described short-term findings, with no studies examining the long-term ramifications of the intervention. Approximately half of the studies included interviews with both women and their male partners. In-depth interviews were the most commonly used method for data collection; however, few studies used focus group discussions (the data collection methods are detailed in Table S1 in Multimedia Appendix 1 [8,26-32,34,36-39]). A summary of the characteristics of the 14 included studies is shown in Table 1 (see Multimedia Appendix 1 [8,26-32,34,36-39] for the detailed characteristics of selected studies). Table 2 provides summary of quality scores for selected articles based on Critical Appraisal Skills Programme checklist. Table S2 in Multimedia Appendix 1 provides details for each of the CASP questions and answers for each paper. Table S3 illustrates each paper by thematic coding - positively transformational, negatively transformational and non-transformative. Table S4 and S5 illustrate end-user involvement from each intervention and data collection methods.

**Table 1:** Characteristics of selected studies.

Study	Journal	Description of mHealth^a^ intervention	Primary objective	Sample	Key findings on gender relations
Alam et al [26]; Bangladesh	International Journal of Environmental Research and Public Health	Provided women with nutrition counseling, support, and information for home gardens and an unconditional cash transfer delivered on a mobile platform	To assess the feasibility and acceptability of the intervention that aims to improve the health of women and children in rural Bangladesh	Qualitative: 20 women and 6 project workers; quantitative: 58 women	Positive transformative: increased spousal communication, further enhanced by mobile phone (received from the project), and cash transfer strengthened independent financial decision-making by women, as well as joint financial decision-making Nontransformative: some women were not free to go to the market to withdraw funds or open a mobile banking account
Alam et al [27]; Bangladesh	JMIR mHealth and uHealth	Pregnant women, new mothers, and their family members accessed weekly voice or SMS text messages and used a 24-hour hotline to contact physicians who provided support on maternal and child health care	To describe the experiences of subscribers and the perceptions of physicians who provided consultations through the *Aponjon* service, focusing on access, acceptability, usability, benefits, and challenges	Qualitative: 8 women, 8 husbands of female subscribers, and 11 medical physicians; quantitative: 3894 subscribers	Positive transformative: increased women’s autonomy in seeking health services; women were not as reliant on men to arrange medical advice or appointments; increased involvement of male partners in health care, resulting in informed decision-making and increased joint health-related decision-making
Atukunda et al [28]; Uganda	AIDS and Behavior	SMS text messages were sent to nominated social support persons of individuals who were HIV positive to help adherence to antiretroviral treatment	To examine individual characteristics and sociocultural dynamics that explain trends in social support and adherence to an SMS text message–based antiretroviral intervention	Qualitative: 10 social supporters; quantitative: 63 participants who were HIV positive and 45 patient-identified social supporters	Positive transformative: improved relationships between participants, particularly if the support person was of a different gender Negative transformative: SMS text messages were sometimes a trigger for relationship problems; the response to the intervention was highly sensitive to existing relationship issues, with support person efforts being perceived negatively, particularly if the support person was the married partner
Brinkel et al [29]; Ghana	Tropical Medicine and International Health	Parents or caregivers accessed health information via an mHealth interactive voice response system to support them in caring for children who were sick	To evaluate user experiences with the interactive voice response system	Qualitative: 37 mothers; quantitative: 37 mothers	Positive transformative: increased women’s health-related knowledge, thus increasing their decision-making ability to make informed decisions regarding the health of their children
Brown et al [30]; Kenya	AIDS and Behavior	Automated SMS text messages were sent to new mothers to notify them of infants’ HIV test results and when infants who were HIV negative were eligible for retesting	To evaluate mothers’ experiences receiving HIV Infant Tracking System–enhanced early infant diagnosis services (acceptability, benefits, and areas for improvement)	Qualitative: 137 women	Positive transformative: increased women’s autonomy in seeking health services because of reduced financial costs, and travel time increased male involvement Negative transformative: reinforced gender divide for women who were illiterate as it increases reliance on the husband to read the message Nontransformative: women’s burden of work and competing responsibilities, and limited resources made it difficult to attend the clinic
Campbell et al [31]; Uganda	AIDS and Behavior	SMS text messaging–based intervention that sent messages to individuals who were HIV positive requesting a return to the clinic after abnormal test	To document the experiences of participants who were HIV positive regarding the SMS text messaging–based intervention in rural Uganda and propose a framework for acceptance of mHealth apps	Qualitative: 43 women and men who were HIV positive	Positive transformative: new means of engaging partners in communication; SMS text messages fostered a sense of closeness and appreciation of emotional support from the partner
Decker et al [32]; Kenya	BMJ Global Health	Women at risk of IPV^b^ used the myPlan app, a safety decision-making and planning mHealth app tailored to the Kenyan context for prevention and response to gender-based violence	To evaluate the efficacy of the app on safety and health outcomes of the myPlan app and intervention	Qualitative: 30 women; quantitative: 352 (n=177 intervention and n=175 control in a 2-arm RCT^c^)	Positive transformative: increased women’s knowledge on safety and rights concerning IPV; enhanced feelings of confidence and resilience; and enabled women to make informed decisions related to their safety, mitigate violence, and deescalate potentially harmful situations with their partners
Hazra et al [33]; India	Journal of Health Communication	Voice messages sent to husbands covering topics such as antenatal care, postnatal checkups, early initiation of breastfeeding, clean cord care, and delayed bathing	To examine whether the distribution of information on maternal and child health to husbands would enhance men’s knowledge and result in the adoption of healthy behaviors	Qualitative: 10 male participants and their wives and 2 FGD^d^ with health care workers; quantitative: 881 husbands	Positive transformative: increased male knowledge of women’s health, thus increasing informed decision-making and communication between couples Negative transformative: reinforcement of traditional gender roles as men alone were provided messages and did not always share information with female partners
Huda et al [34]; Bangladesh	JMIR mHealth and uHealth	Pregnant women and new mothers were provided with a free mobile device and received interactive voice messages, direct nutrition counseling from a call center, and an unconditional cash transfer via mobile banking	To determine the feasibility, acceptability, and appropriateness of the intervention designed to improve nutrition during pregnancy and the first year of life for women and children in rural Bangladesh	Qualitative: 21 participants; quantitative: 340 pregnant or recently delivered women	Positive transformative: increased women’s ability to translate health-related information into practice; increase in spousal communication Nontransformative: traditional duties and gender-based roles were noted as a barrier to access (restricted movement outside the house and lack of ability to go to the marketplace to access cash)
Ilozumba et al [8]; Uganda	JMIR mHealth and uHealth	SMS text messaging platform designed to provide participants with information regarding upcoming antenatal care visits and recommendations on reproductive health practices	To outline the assumptions of the program designers and contrast their assumptions with empirical data to better understand facilitators and barriers related to the outcomes of the program	Qualitative: 15 female participants, 11 male participants, FGDs with 50 village health team members, and interviews with 6 health service providers	Positive transformative: increased male involvement in maternal health decision-making (men own phones); increased women’s ability to demand health services, enhancing joint health-related decision-making Negative transformative: male partners were noted as a barrier by some, as they were not intended primary beneficiaries, thus reinforcing gender differentials in women’s decreased levels of mobile phone ownership and lower rates of female literacy
McBride et al [36]; Vietnam	Journal of Public Health	mMom is an mHealth platform that sends SMS text messages to improve women’s health during pregnancy by encouraging their use of health services	To determine whether implementation of a low-cost mHealth intervention could increase ethnic minority women’s access to maternal, newborn, and child health services	Qualitative: 60 female participants and 8 individual interviews with community health workers	Positive transformative: increased husbands’ interest and engagement in maternal and infant health, increased health-related joint decision-making, and enhanced women’s empowerment to make informed decisions about health care
Nyemba-Mudenda and Chigona [37]; Malawi	Information Technology for Development	The Mobile System for Safe Motherhood is a toll-free hotline, interactive voice response, and SMS text messaging system designed to provide pregnant women with maternal health-related information, tips, and appointment reminders	To assess whether the use of mobile phones in maternal health can enable capability outcomes and outline the factors that facilitate and restrict the outcomes from being enabled	Qualitative: 46 (26 female participants, 4 community volunteers, 4 midwives, 4 health facility managers, and 4 stakeholders; 32 IDIs^e^ and 2 FGDs)	Positive transformative: women empowered by health information gained the support of husbands, spousal communication improved as they listened to messages on the shared phone, and male knowledge of and involvement in maternal care and support of women’s access to health services increased Negative transformative: increased arguments with male partners; women could not adapt all recommendations as gender roles prohibited the woman from resting when pregnant
Shelus et al [38]; Kenya	International Perspectives on Sexual and Reproductive Health	mHealth app designed to assist women in tracking their menstrual cycles to plan or prevent pregnancy	To explore women’s experiences with using the CycleBeads app and how this experience varied based on how the participant learned about the app	Qualitative: 28 female app users; quantitative: 185 female app users	Positive transformative: increased women’s knowledge of fertility and tracking of the menstrual cycle, enhanced confidence in preventing pregnancy, improved communication with their sexual partner, and increased health-related joint decision-making
Velloza et al [39]; Kenya	MHealth	Tablet-based app developed for use by providers during consultations with couples who were HIV serodiscordant, which derives data from, women via SMS text messages to assist health workers in providing counseling on safe conception options	To assess the acceptability and feasibility of the Safer Conception Intervention for Partners app	Qualitative: 19 couples who were HIV serodiscordant and 5 health care providers; quantitative: 74 couples who were HIV serodiscordant	Positive transformative: increased women’s knowledge, which enabled more informed decisions regarding health, strengthened communication with partners, and increased health-related joint decision-making between partners Negative transformative: a report of verbal and physical abuse was related to a misconception about the source of SMS text messages

^a^mHealth: mobile health.

^b^IPV: intimate partner violence.

^c^RCT: randomized controlled trial.

^d^FGD: focus group discussion.

^e^IDI: in-depth interview.

**Table 2:** Summary of quality scores for selected articles based on Critical Appraisal Skills Programme checklist (N=14).

Item number	Items	Articles, n (%)
1	Clear statement of aims	14 (100)
2	Appropriate methodology applied	14 (100)
3	Appropriate research design	14 (100)
4	Appropriate recruitment strategy	14 (100)
5	Appropriate data collection methods	11 (79)
6	Reflexivity noted by researchers	1 (7)
7	Ethical issues are taken into consideration	10 (71)
8	Sufficiently rigorous data analysis	13 (93)
9	Clear statement of findings	14 (100)
10	Discusses the value of research	14 (100)

### Measuring Influence on Gender Relations

None of the studies we reviewed specifically appraised gender relationships from the outset. However, 21% (3/14) of studies examined relationships between women and men: the study by Campbell et al [29] on the acceptance of an SMS text message–based intervention for people living with HIV asked questions about how the intervention affected relationships; the examination by Decker et al [30] of a safety decision-making app for women at risk of IPV studied relationship quality and changes in self-efficacy; and the study by Hazra et al [33] considered the change in the relationship between husband and wife when the husband was the recipient of SMS maternal and child health voice messages. The remaining 79% (11/14) of studies uncovered findings on gender relations through the course of the intervention or postprogram evaluation and did not assess long-term changes that occurred because of the intervention. Half of the evaluated studies interviewed women only, and the other half interviewed women and men (sexual partners and spouses).

Half of the studies included in this review interviewed both women and men. Recent studies have highlighted the value of interviewing both partners as responses can often differ [40,41]. The inclusion of both partners in the interviews is a *hotly debated* topic in family studies. Dyadic interviews can lead to richer information and more evidence gathered as couples feed off each other, provide more information, and offer different perspectives. However, one partner can dominate the discussion and may limit the freedom of the other to respond truthfully. Using participant observation and observing the interaction between men and women in the interview itself may provide results in decision-making, gender relations, and negotiations between couples.

### Positive Transformative Influence on Gender Relations

#### Spousal Communication

This review of the literature revealed several positive ways in which mHealth interventions could transform gender relations. Our findings showed improved spousal communication on an everyday basis when learning together and regarding health-related information. Several studies reported an increase in everyday communication [26,31]. During the postprogram analysis, the study by Alam et al [26] assessing the feasibility of a nutrition intervention that used mHealth and provided women in rural Bangladesh with a mobile phone showed that daily communication with their spouse increased. Women spoke of the benefits of communication: “I can call [my husband] in case of any problem using this mobile phone. I have been benefited as my husband has one mobile phone that he always keeps with him and carry wherever he goes. Now, if my husband goes outside, he calls me in my phone if necessary, isn’t it good for me*?*” [26]. In a trial of an SMS text messaging–based intervention for people living with HIV in rural Uganda, Campbell et al [31] also found that the intervention fostered a new means of engaging partners to communicate regularly by phone.

When the mHealth intervention contained a training component, gender relations transformed as couples spent time learning together (agricultural and nutrition training), which would not usually occur in many countries because of the gendered division of labor. The study by Alam et al [26] observed that women worked with their husbands to create homestead gardens, fostering collaboration and communication. Spousal communication increased as couples discussed the health information provided by the mHealth intervention [33,36,37]. In India, Hazra et al [33] found that male participants, recipients of voice messages on maternal and child health, said they would discuss how to follow health-related instructions with their wives, as per the intervention’s recommendations. One of the fathers would record the messages and play them back to discuss healthy practices with his wife [33]. In Vietnam, McBride et al [36] also found that ethnic minority women shared SMS text messages on maternal and child health with their husbands, thus enhancing communication between couples. According to the study by Nyemba-Mudenda and Chigona [37], couples in Malawi would read SMS text messages and listen to interactive voice messages on maternal health together, share and discuss information, and report enhanced communication on health-related topics. In addition to discussing health information, women in several studies reported an increased ability to communicate openly with male partners on sexual and reproductive health topics [38,39]. Increased communication between partners improved their ability to cooperatively use contraceptive choices [38,39]. In Kenya, an mHealth app called CycleBeads was designed to assist women in tracking their menstrual cycles to plan for or prevent pregnancy [38]. Women using the app described improved communication with their sexual partners, saying, “He thinks I don’t want to have sex with him. But after showing him this application, even he knows it’s unsafe to have unprotected sex” [38]. Another study in Kenya used SMS text messages to promote safer conception for couples who were HIV serodiscordant and reported similar outcomes, affirming that male reproductive health knowledge improved mutual communication with their partners regarding conception strategies [38].

#### Emotional Support From Partner

The literature also showed that mHealth interventions enhanced emotional support between couples [30-33,37]. Brown et al [30] presented findings from their SMS text messaging–based intervention in Kenya, which aimed to improve the early diagnosis of infants who were HIV positive. SMS text messages sent to women provided new opportunities for male partners to communicate emotional support to their partners. The study by Campbell et al [31] found that individuals who were HIV positive in rural Uganda stated that the SMS text messages fostered a sense of closeness and appreciation of emotional support from their partners. Interaction with partners and family members altered; when one husband was asked whether the messages brought any changes to his relationship with his wife, he replied that they fostered a sense of trust: “We got to love each other more...we keep communicating on the phone...and this change of heart started with the message” [31]. In Malawi, women using an SMS text messaging and toll-free hotline on maternal health said that the discussion of information provided a sense of support from their husbands [37]. Decker et al [32] reported that the myPlan mHealth app in Kenya, an interactive tool that survivors of IPV can use to aid in safety decision-making, reduced decisional conflict within relationships. Women became more resilient and learned to mitigate violence and abuse from their partners [32]. Modes of spousal communication were transformed as women learned how to de-escalate potential violence: “now when he comes home, I study his mood so that I know how to handle him in order to avoid the chaos” [32].

#### Decision-making

Numerous studies revealed that men were becoming more involved in maternal and child health, which is traditionally seen as a domain of women [30,35,36]. The study by McBride et al [36] revealed that men in Vietnam exhibited a new interest in maternal and child health and supported their wives in attending neonatal health services. The study by Ilozumba et al [35] reported similar findings, stating that men’s involvement had an unintended positive consequence in Uganda, and by receiving SMS text messages, they became more involved in maternal health care. By participating in mHealth interventions, men increased their health-related knowledge associated with women’s and children’s health [33,42]. This knowledge enhanced informed decision-making on the part of men and fostered health-related decision-making between partners [26,33,35,36,42]. Owing to the gendered divide in mobile phone ownership, several studies reported having to enroll men in maternal and child health programs as women did not own phones [33,35]. In India, Hazra et al [33] ascertained that mHealth messages sent only to men improved joint decision-making with their partners. Alam et al [42] also observed enhanced health-related joint decision-making between couples in Bangladesh following the use of the *Aponjon* maternal and child health care hotline [42]. In Uganda, when men enrolled in a maternal SMS text messaging–based intervention intended for women, their increased involvement led to an increase in joint health-related decision-making [35].

#### Increased Male Involvement: Resource Allocation

On the basis of gender roles, men are often the primary household decision-makers and have greater access to resources. However, male partners provided additional financial support to women when provided with information regarding women’s and children’s health [29,30,36,37]. The study by Alam et al [26] combining nutrition and agricultural counseling with an unconditional cash transfer reported that women made decisions, either on their own or in conjunction with their husbands, about how the cash transfer would be spent, thus altering gender roles. A phone-based intervention supporting parents to care for children who were sick in Ghana was perceived as a mechanism of reducing the barrier of women not having control over financial resources and not making decisions without their husbands’ support [29]. The intervention provided women with information and allowed them to participate in health-related decision-making [29].

In rural Bangladesh, 14% (2/14) of mHealth studies provided unconditional cash transfers to women and revealed barriers to receiving cash. The obstacles included women having no national identity card to open a web-based banking account or not being able to go to the market (prohibited or culturally unacceptable) to withdraw money. However, the studies by Alam et al [26] and Huda et al [34] found that women received support from husbands, male family members, or children to open accounts or collect money from mobile banking agents in the marketplace. The funds received through this program provided women with cash that they could spend on food, medicine, and other supplies, with most women deciding how to spend the money themselves [34]. Women and men cooperated and made decisions jointly about expenditure, whereas, previously, they would not necessarily have had such inputs. These mHealth interventions demonstrate that they can economically empower women, overcome obstacles, use mobile banking, and access financial resources.

#### Autonomy in Seeking Health Information and Access to Services

The literature showed that, overall, mHealth interventions have the capacity to increase women’s autonomy in seeking access to health care and improve access to health information. mHealth interventions can reduce gender-based barriers, such as spousal permission, lack of freedom of movement, the necessity for male accompaniment, and requiring financial support [26,29,30,35,37-39,42]. Studies suggest that when women have increased health-related knowledge, they become more empowered to demand essential health services and quality care [35,37].

In Bangladesh, based on interviews with both women and men after the intervention, Alam et al [42] found that when women could access an mHealth hotline independently, it increased their autonomy in seeking health services. Women found the hotline convenient, could act independently, and make calls on their own; they were no longer reliant on men to arrange medical advice or appointments. In Kenya, where women used the myPlan IPV prevention app, this was also the case as women reported building resilience and confidence in discussing IPV and gaining support, knowledge, and access to the available services [32]. Men are often the key decision-makers in rural Ghana, with women not always having access to reliable health information and services or control over household resources. Women participating in an IVR system in Ghana reported that the IVR provided them with trustworthy information, which enabled them to have more control over their health care and that of their children and empowered them to make independent health-related decisions [29].

#### Self-efficacy

Our findings also revealed that mHealth programs increased women’s independence in seeking access to health services and led to positive changes in women’s self-efficacy. Despite the low rates of female phone ownership, Nyemba-Mudenda and Chigona [37] reported that women gained self-confidence and skills by communicating via mobile phones, reducing the gendered digital divide [37]. In gaining health-related knowledge, women found that their confidence was enhanced, as was their capacity to put this knowledge into practice [34,37-39,43]. Women in Vietnam who participated in an SMS text messaging–based program on maternal and child health felt empowered by this newfound knowledge, made informed decisions about health care, and were more confident in their interaction with community health workers [36]. In Malawi, women were provided with maternal health knowledge and support and subsequently empowered to request a health service or attention from a health care worker; in contrast, before the intervention, they would “settle for whatever assistance was given” [37]. In Ghana, Brinkel et al [29] found that health information contributed to empowerment, altered gender relations, and challenged women’s low decision-making abilities. In Kenya, an IPV safety app motivated a woman to sell clothes [32]. As a result of earning additional income, the woman could buy food for her children and no longer had to wait for her partner, thus providing autonomy in financial decision-making, when previously her husband would beat her if she asked for money for food [32].

### Negative Transformative Influence on Gender Relations: Relational Conflict and Decision-making

Despite the improvements discussed so far, mHealth interventions have the potential to exacerbate or ignore the persistent gender-based barriers that women face [30,31,33,35,37]. In Uganda, the study by Atukunda et al [28] reported an increase in conflict between couples while taking part in an SMS text messaging–based HIV support program. The trial used SMS text messages sent to a nominated social support person of an individual who was HIV positive and aimed to improve antiretroviral adherence [28]. Although the authors noted that the relationship turmoil was not a direct result of the intervention itself, the SMS text messages may have been a trigger as relations between some of the couples either stalled or became turbulent, exemplified by feelings such as lack of trust, unsupportive behavior, resentment, suspicions of infidelity, stigma, or fear of disclosure of HIV status. One of the women reported, “He shouts at me for constantly asking him about his medicines every day, so I stopped asking about them. He doesn’t listen to me at all and says I nag him” [28]. The intervention proved to be a catalyst, exacerbating relationship problems, particularly if the support person was the spouse of the person who was HIV positive. Velloza et al [39] recounted an instance of verbal and physical abuse in an SMS text messaging intervention in Kenya, which supported a safer conception for couples who were serodiscordant and living with HIV when a male partner believed that the SMS text message was from a former partner. In Malawi, community attitudes toward a maternal and child mHealth intervention were suspicious as they thought the intervention was “a satanic gimmick to get blood from pregnant mothers’ bodies and kill the babies,” which led to conflict between husbands and wives [37]. Some men forbade or stopped their wives from using the service, forcing them to leave the intervention [37]. Women would obey their husbands out of fear and respect or run the risk of being forced out of the house [37].

### Nontransformative Influence on Gender Relations

#### Gender Gaps in Literacy

Evidence also indicates that mHealth programs could be nontransformative and reinforce gender-based inequalities. An mHealth trial in Kenya used SMS text messages to remind mothers to take their babies to the clinic for HIV testing; however, some women were illiterate and unable to read or understand the SMS text messages [30]. When literacy rates are lower among women, reliance on SMS text messages reinforces gender divisions and women’s dependence on husbands to enable access to information.

#### Men as Gatekeepers of Technology and Information

A maternal and child health app in India sent SMS text messages to only men, reinforcing the role of men as gatekeepers of information and decision-makers in the family [33]. Although some men shared and discussed the information, a substantial number of men did not. Some men stated that they “did not feel the need” to discuss the messages; others said they were busy at work or just not interested in such messages, which was thought of as women’s business and knowledge that the mother should already know [33]. One of the studies indicated that low female ownership of mobile phones could reinforce reliance on men and the conduit a woman must go through to obtain mHealth information. In Uganda, men were enrolled to receive SMS text messages on maternal and child health targeted at women; although for some, this increased male involvement in reproductive health decisions, it also proved to be a barrier for some women. A Ugandan woman enrolled in a study reported not receiving any antenatal care until the seventh month of pregnancy as “her husband had not given her permission,” illustrating that her husband had been a barrier to her seeking health services [35].

## Discussion

### Principal Findings

We reviewed the impact of mHealth interventions on gender relations in LMICs based on studies published between 2013 and 2020. Our results demonstrate that mHealth interventions have the potential to improve women’s health, enhance digital literacy, positively affect women’s empowerment, and enhance gender relationships. The findings also revealed that mHealth programs could reinforce gender divisions, exacerbate domestic conflict, and reinforce the dominance of men as key decision-makers and gatekeepers of knowledge and mobile technology. Gender-based digital divide, women’s lack of access, and digital literacy have been well documented. However, despite the increase in the use of mHealth apps, most studies continue to focus on the feasibility and acceptability of such interventions, with none of the reported studies explicitly assessing the positive or negative impact of the intervention on gender relations.

Despite these data limitations, several key findings emerged. The studies revealed that mHealth interventions could positively affect spousal relationships and enhance communication and decision-making on health-related topics. Messages on maternal and child health sent via mHealth platforms to either the woman or man’s phone were listened to and shared between couples. The new knowledge gained was discussed, and communication between couples improved. Several studies reported starting a dialogue on sexual and reproductive health, topics traditionally seen as “women’s business.” In Kenya and Uganda, mHealth programs targeting people living with HIV found that communication and emotional support between couples were enhanced [31,39]. Another program in Kenya, targeting safety for women at risk of IPV, was reported as being transformative for relationships as women gained skills to communicate with their partners in new ways and mitigate the risk of IPV [32].

The review found that mHealth interventions improved men’s health-related knowledge associated with women’s and children’s health, and this knowledge increased informed health-related decision-making on the part of the men and fostered health-related decision-making between partners. Men either received the messages or were the owners of the phone that their partners needed to access, and therefore, the sometimes unintentional inclusion of the husband had the positive effect of accelerating access to health care. Our findings also suggest that mHealth interventions have the ability to increase male partners’ understanding of women’s health, thus enabling them to act as facilitators to increase women’s access to health services and information by providing either financial or emotional support. Mobile phone ownership is still low in some parts of Malawi and particularly so for women. Many women in this intervention relied on their husbands’ phones to receive the messages, with this male involvement being described as a “paradigm shift” [37].

Engaging men in mHealth interventions can increase their ability to make informed decisions related to their female partners’ or children’s health [44]. Furthermore, male participation in mHealth interventions can increase joint health-related decision-making between partners and enhance health-related communication, translating into better health practices. Participants in a mobile phone–based messaging service in maternal and newborn health in Afghanistan reported that involving fathers was beneficial, and joint decision-making between wives and husbands increased [45]. The “Super Abbu” (Super Dad) pregnancy and infant hotline in Pakistan was inundated with calls from fathers, with approximately 40,000 calls within the first 2 months, illustrating the need to include fathers and engage men for optimal health outcomes for women and children [46].

In many households, men are the primary household decision-makers and have greater access to income. Several studies reported changes in power dynamics over financial matters, particularly if the intervention incorporated cash transfers [26,34]. Women gained financial autonomy and control over income as recipients of cash transfers. Women were no longer as reliant on men for financial support for health and nutrition decision-making, enhancing control over financial resources and input into decisions regarding expenditure.

This review established that mHealth interventions can increase women’s autonomy in seeking access to health care and improve access to health information. mHealth interventions can reduce gender-based barriers, such as requesting financial support, gaining spousal approval, and the need for male accompaniment. The literature also suggests that women’s participation in mHealth interventions can increase women’s autonomy in accessing health services and health information. Furthermore, these interventions can empower women to translate their knowledge into practice. Thus, mHealth interventions can enhance women’s active care-seeking behavior, increase their ability to adopt healthier practices, and enhance their confidence to demand better quality care. In Nepal, research has found that telemedicine could overcome gender-based barriers to accessing health services in rural Nepal [47]. These conclusions concur with our findings, revealing that women’s participation in mHealth interventions could increase women’s autonomy in seeking health services through reduced travel restrictions, time, and financial costs [47]. Our findings also revealed that women reported an increased sense of self-efficacy with health-related knowledge and were more empowered and confident in their decision-making ability [44]. Increasing evidence suggests that digital health positively influences health equity [20].

Despite these positive impacts, we reported on several gender-based barriers. mHealth interventions can have an adverse effect, reinforcing the digital divide and upholding men as gatekeepers of information and sole decision-makers. Interventions can reflect and reinforce existing gender-based inequities such as the digital divide or hinder access to resources or information. mHealth can emphasize women’s reliance on men to access technology [44]. A recent study on an mHealth maternal nutrition intervention from Burkina Faso revealed that although the researchers did not focus their research on gender at first, it proved to be highly relevant to their study [48]. Mothers who took part in the nutrition intervention stressed that they were “not empowered to make nutrition-based decisions that incur costs...nutrition-related request can spark marital strife” [48]. This illustrates the risk that mHealth interventions can pose in increasing women’s reliance on men for economic resources.

When mHealth interventions strengthen the role of men as gatekeepers, controlling access to mobile phones and information received, as women have lower literacy rates than men, this increases a woman’s dependency on men and can lead to conflict. Previous studies have shown that mHealth interventions can lead to increased tension between couples and domestic disputes and precipitate IPV [16,49]. However, few studies have evaluated or reported on its potential harm. Unintended consequences can occur when gender dynamics are not assessed. An mHealth intervention promoting contraceptive use in rural Bangladesh noted an increase in reports of IPV linked to participation in the program, a conflict that may have resulted from women receiving phone calls from an unknown number [49]. Another study from Ghana assessed the impact of family planning on gender relations and reported increased tension in relationships, with men reporting that they feared that their wives would be unfaithful as they now used contraception [50]. These findings highlight the need to monitor the intended and unintended consequences of mHealth interventions on gender relationships.

The findings of this review have several limitations. Qualitative data are largely context specific, making these findings nongeneralizable to broader settings. Similarly, as gender relations are highly dependent on sociocultural factors, generalizability and transferability may be further limited. In addition, it is possible that some literature was overlooked as the search was limited to journal articles published in English and to those available electronically. Furthermore, this search did not include any gray literature or unpublished sources. Despite these limitations, our research team applied a comprehensive and robust search strategy to enhance the rigor of this review.

### Program

Given the rapid, persistent upscale of mHealth interventions in low- and middle-income settings, it is imperative for intervention teams to design these interventions while considering their impact on health equity, power dynamics, and gender relations. Efforts should be made to promote positive impacts while mitigating negative effects. To promote the positive impact of mHealth interventions on gender relations, rigorous formative research is needed to assess the context-specific requirements of the intervention and the participants. The key to this is involving the end user to inform and co-design interventions to ensure that they are appropriate, feasible, and safe in the context in which they are implemented. Thorough monitoring and evaluation throughout the course of the intervention are also recommended. Researchers must design gender-transformative mHealth interventions to truly affect change and not exacerbate existing gender inequalities [12,51]. Future research is required to fill the evidence gaps in gender and mHealth, acknowledging that women are not passive beneficiaries and need to actively participate and be empowered by mHealth interventions. These interventions require rigorous assessment from a gender perspective, from design and implementation to evaluation, to explore their impact on women and men from the outset.

## Appendix

Multimedia Appendix 1 Supplementary material.

## Abbreviations

IPV: intimate partner violence
IVR: interactive voice response
LMIC: low- and middle-income country
mHealth: mobile health
PRISMA: Preferred Reporting Items for Systematic Reviews and Meta-Analyses
PROSPERO: International Prospective Register of Systematic Reviews
