# Supplementary material for: The Role of mHealth Interventions in Changing Gender Relations: Systematic Review of Qualitative Findings
Source: JMIR Hum Factors. 2022 Jul 21;9(3):e32330. doi: 10.2196/32330 (PMC9353673; doi:10.2196/32330)
Supplement: Multimedia Appendix 1 [file humanfactors_v9i3e32330_app1.doc]

**Multimedia Appendix 1**

**Table S1. Detailed characteristics of selected studies.**

| **Author(s)**  **Year**  **Journal**  **Country** | **Description of mHealth intervention** | **Primary objective** | **Design** | **Sample** | **Key findings on gender relations** |
| --- | --- | --- | --- | --- | --- |
| **(1) Alam *et al.* 2020 (26)**  ***International Journal of Environmental Research and Public Health***  **Bangladesh** | A multi-component intervention that provides women with nutrition counselling, support and information for home gardens and an unconditional cash transfer, delivered on a mobile platform, with the aim of improving the health of women and children in rural Bangladesh. | To assess the feasibility and acceptability of the intervention, which combined a cash transfer with agricultural training and nutrition counselling for the participants and project workers. | Single group, post-test using mixed methods | Qualitative:  20 women, 6 project workers  Quantitative:  58 women | Positive transformative:  Increased spousal communication, further enhanced by mobile phone (received from the project), cash transfer is given to women strengthened independent financial decision making as well as joint financial decision making, the new knowledge on nutrition and home- gardening fostered an increase in communication and cooperation between wife and husband.  Non-transformative:  Some women were not free to go to the market to withdraw funds or open a mobile banking account. |
| **(2) Alam *et al.* 2019 (27)**  ***JMIR mHealth and uHealth***  **Bangladesh** | Pregnant women, new mothers, and their family members can access weekly voice or SMS messages and utilise a 24-hour hotline to contact doctors who provide support on maternal and child health care. | To describe the experiences of subscribers and the perceptions of doctors who provided consultations through the *Aponjon* service, focusing on access, acceptability, usability, benefits, and challenges. | Single group, pretest design, post-test using mixed methods. | Qualitative:  16 families: 8 women subscribers, 8 husbands of female subscribers, and 11 medical doctors (9 females and 2 males)  Quantitative:  3894 subscribers to *Aponjon* | Positive transformative:  Increased women's autonomy in seeking health services empowered them to discuss maternal and child health issues; women were not as reliant on men to arrange medical advice/ appointments. Increased involvement of male partners in health care resulting in informed decision-making and increased joint health-related decision making. |
| **(3) Atukunda et al.**  **2017**  **(28)**  ***AIDS and Behavior***  **Uganda** | SMS notifications are sent to nominated social support persons of HIV individuals to help adherence to antiretroviral treatment. | To examine individual characteristics and socio-cultural dynamics that explain trends in social support and adherence to a SMS based antiretroviral intervention. | Randomised control trial pilot study, Dual group, pretest design using mixed methods. | Qualitative:  10 social supporters  Quantitative:  63 HIV positive participants (randomised into 2 types of SMS reminder and control group) and 45 patient identified social supporters. | Positive transformative:  Improved relationships between participants, particularly if the support person was of a different gender.  Negative transformative:  SMS messages were sometimes a trigger for relationship problems; the response to intervention was highly sensitive to existing relationship issues, with support person efforts perceived negatively, straining relationships, and fostering feelings of resentment, particularly if the support person was the married partner. |
| **(4) Brinkel *et al.* 2017**  **(29)**  ***Tropical Medicine and International Health***  **Ghana** | Parents or caregivers can access health information via a mHealth interactive voice response system to support them in caring for sick children. | To evaluate user's experiences with the interactive voice response system (adherence, usability, perceived opportunities, and barriers) | Single group, pretest design, post-test using mixed methods. | Qualitative:  37 mothers (focus group)  Quantitative:  37 mothers | Positive transformative:  Increased women's health-related knowledge, thus increasing their ability to make informed decisions regarding the health of their children. The information empowered women and gave them more control and greater input in decision making about health care for themselves and their children. |
| **(5) Brown *et al.***  **2019**  **(30)**  ***AIDS and Behavior***  **Kenya** | Automated SMS messages sent to new mothers to notify them when their infants' HIV test results are available and when HIV-negative infants are eligible for retesting. | To evaluate mothers' experiences receiving HIV Infant Tracking System-enhanced early infant diagnosis services (acceptability, benefits, and areas for improvement) | Dual group, post-test, embedded in cluster randomised control trial.  Qualitative methods. | Qualitative:  137 women. | Positive transformative:  increased women's autonomy in seeking health services due to reduced financial costs and travel time increased male involvement (financial support and encouragement), resulting in facilitation of earlier clinic appointments.  Negative transformative:  Reinforce gender divide if women are illiterate as increases reliance on the husband to read the message, and husband not always around.  Non-transformative:  Women's burden of work and competing responsibilities, and limited resources made it difficult to attend the clinic. |
| **(6) Campbell *et al.***  **2017**  **(31)**  ***AIDS and Behavior***  **Uganda** | SMS based intervention that sent messages to HIV positive people requesting a return to the clinic after abnormal laboratory test (low CD4 count). | To document HIV+ participants experience in an SMS based intervention in rural Uganda and propose a framework for technology acceptance for mHealth applications targeting low-literacy populations in resource-limited settings. | Single group, post-test clinical trial. | Qualitative:  43 HIV positive women and men | Positive transformative:  new means of engaging partners to communicate, and SMS fostered a sense of closeness and appreciation of emotional support from the partner. |
| **(7) Decker *et al.* 2020**  **(32)**  ***BMJ Global Health***  **Kenya** | Women at risk of IPV utilise myPlan app, a safety decision making and planning mHealth app that is tailored for the Kenyan context for prevention and response to gender-based violence. | To evaluate the efficacy on safety and health outcomes of the myPlan app and intervention. | Dual group, pre and post-test, 2 arm RCT, mixed methods. | Qualitative:  30 women at risk IPV  Quantitative:  352 (n=177 intervention, n=175 control in 2 arm RCT) | Positive transformative:  Increased women's knowledge on safety and rights concerning IPV, enhanced feelings of confidence and resilience and enabled women to make informed decisions related to their safety, mitigate violence, and deescalate potentially harmful situations with their partners. |
| **(8) Hazra *et al.***  **2018**  **(33)**  ***Journal of Health Communication***  **India** | Voice messages are sent to husbands covering topics such as antenatal care, postnatal checkups, early initiation of breastfeeding, clean cord care, and delayed bathing. | To examine whether the distribution of information on maternal and child health to husbands would enhance the man's knowledge, trigger discussions with wives/family members and result in the adoption of healthy behaviours. | Dual group, post-test using mixed methods | Qualitative:  10 male participants and their wives, 2 FGD with healthcare workers  Quantitative:  881 husbands (428 intervention / 453 control) and 956 women (478 intervention and 478 control) | Positive transformative:  Increased male knowledge of women's health, thus increasing informed decision-making and communication between couples.  Negative transformative:  Reinforcement of traditional gender roles as men alone were provided messages and did not always share information with female partners, strengthening men's role as the sole decision-maker and gatekeepers of information, husbands did not share information as not interested or maternal health information not seen as men's business. |
| **(9) Huda *et al.***  **2018**  **(34)**  ***JMIR mHealth and uHealth***  **Bangladesh** | Pregnant women and new mothers were provided with a free mobile device through which they received interactive voice messages, direct nutrition counselling from a call centre, and an unconditional cash transfer via mobile banking | To determine the feasibility, acceptability, and appropriateness of a multifaceted intervention package designed to alter perceptions on nutrition during pregnancy and the first year of life for women and children in rural Bangladesh. | Single group, pretest and post-test design using mixed methods | Qualitative:  21 participants (7 pregnant women, 7 women recently given birth, 5 husbands and 2 mothers-in-law)  Quantitative:  340 pregnant or recently delivered, lactating women. | Positive transformative:  Increased women's ability to translate health-related information into practice, increase in spousal communication as new knowledge shared with husband, navigating the mobile banking app also enhanced communication and cooperation between wife and husband.  Non-transformative:  Traditional duties and gender-based roles were noted as a barrier to access (restricted movement outside the house and lack of ability to go to the marketplace to access cash, did not have an ID card to open a bank account). |
| **(10) Ilozumba *et* *al.***  **2018**  **(8)**  ***JMIR mHealth and uHealth***  **Uganda** | SMS platform designed to provide participants with information regarding upcoming antenatal care visits and recommendations on reproductive health practices | To outline assumptions of the program designers and contrast their assumptions with empirical data to better understand facilitators and barriers related to the outcomes of the program. | Single group, retrospective qualitative study | Qualitative:  15 female participants, 11 male participants, FGDs with 50 village health team members and interviews with 6 health service providers. | Positive transformative:  Increased male involvement in maternal health decision making (men own phones), Increased women's ability to demand health services and quality of care, enhancing joint health-related decision making.  Negative transformative:  Male partners were noted as a barrier by some, as they were not intended primary beneficiaries, thus reinforcing gender differentials in women's decreased levels of mobile phone ownership and lower rates of female literacy, consolidating males as decision-makers in matters of reproductive health. |
| **(11) McBride *et* *al.***  **2018**  **(36)**  ***Journal of Public Health***  **Vietnam** | mMom is a mHealth platform that sends SMS messages designed to improve women's health during pregnancy and new motherhood by encouraging their use of maternal and neonatal health services and through increased awareness of risk factors. | To determine whether implementation of a low-cost mHealth could increase ethnicity minority women's access to maternal, newborn, and child health services. | Single group, post-test design qualitative study | Qualitative:  60 female participants (4 FGDs and 30 IDIs) and2 FGD and 8 individual interviews with community health workers. | Positive transformative:  Husbands increased interest and engagement in maternal and infant health, increased health-related joint decision making, enhanced women's empowerment to make informed decisions about health care. |
| **(12) Nyemba-Mudenda *et al.***  **2017**  **(37)**  ***Information Technology for Development***  **Malawi** | The Mobile System for Safe Motherhood (MSSM) is a toll-free hotline, interactive voice response, and SMS system designed to provide pregnant women with maternal health-related information, tips, and appointment reminders. | To assess whether the use of mobile phones in maternal health can enable capability outcomes and outline the factors that facilitate and restrict the outcomes from being enabled. | Single group, post-test design qualitative study | Qualitative:  46 (26 female participants, 4 community volunteers, 4 midwives, 4 health facility managers, 4 stakeholders (32 IDIs and 2 FGDs) | Positive transformative:  Women empowered by health information (self-confidence and expression) and in turn gained the support of husbands (male involvement in maternal care seen as a paradigm shift), spousal communication improved as they listened to messages on the shared phone, increased male knowledge and involvement on maternal care and support women's access to health services.  Negative transformative:  Increased tension and arguments with male partners being a barrier to participate in the intervention; women could not adapt all recommendations as gender roles (such as doing all household chores for extended family) prohibited the woman from resting when pregnant, arguments with husbands over the "satanic" MSSM app and subsequently some men forbade their wives from being part of the intervention, and if they were involved refused to allow them to go to clinic or community volunteer. |
| **(13) Shelus *et* *al.***  **2017**  **(38)**  ***International Perspectives on Sexual and Reproductive Health***  **Kenya** | mHealth application designed to assist women in tracking their menstrual cycles to plan or prevent pregnancy. | To explore women's experiences with using the CycleBeads application and how this experience varied based on how the participant learned about the app. | Single group, pretest and post-test design using mixed methods | Qualitative:  28 female participants  Quantitative:  185 female app users | Positive transformative:  increased women's knowledge on fertility and tracking menstrual cycle, and enhanced confidence on preventing pregnancy, improved communication with their sexual partner and increased health-related joint decision making. |
| **(14) Velloza *et* *al.***  **2019**  **(39)**  ***MHealth***  **Kenya** | Tablet-based application developed for use by providers during consultations with HIV serodiscordant couples which, in part, derives its' data from fertility information and sexual behaviour, sent by women via SMS to assist health workers in providing counselling on safe conception options. | To assess the acceptability and feasibility of the Safer Conception Intervention for Partners application | Single group, post-test design using mixed methods | Qualitative:  19 heterosexual HIV serodiscordant couples and 5 healthcare providers  Quantitative:  74 heterosexual HIV serodiscordant couples | Positive transformative:  Increased women's knowledge, which enabled more informed decisions regarding health, strengthened communication with partners, increased health-related joint decision making between partners.  Negative transformative:  One report of verbal and physical abuse was related to a misconception about the source of SMS messages. |

|  | **Table S2 . Critical Appraisal Skills Programme (CASP) Questions** | | | | | | | | | |
| --- | --- | --- | --- | --- | --- | --- | --- | --- | --- | --- |
|  | Was there a clear statement of aims? | Is a qualitative methodology appropriate? | Was the research design appropriate to address the aims of the research? | Was the recruitment strategy appropriate to the aims of the research? | Was the data collected in a way that addressed the research issues? | Was reflexivity noted by the researchers? | Have ethical issues been taken into consideration? | Was the data analysis sufficiently rigorous? | Is there a clear statement of findings? | Is the value of the research discussed? |
| **Alam *et al.***, 2020 | Yes | Yes | Yes | Yes | Yes | No | Yes | Yes | Yes | Yes |
| **Alam *et al.***, 2019 | Yes | Yes | Yes | Yes | Yes | No | Yes | Yes | Yes | Yes |
| **Atukunda *et al.***, 2017 | Yes | Yes | Yes | Yes | No | No | Yes | Yes | Yes | Yes |
| **Brinkel *et al.***, 2017 | Yes | Yes | Yes | Yes | No | No | Yes | Yes | Yes | Yes |
| **Brown *et al.***, 2019 | Yes | Yes | Yes | Yes | Yes | No | Yes | Yes | Yes | Yes |
| **Campbell *et al.***, 2017 | Yes | Yes | Yes | Yes | Yes | Yes | No | Yes | Yes | Yes |
| **Decker *et al.***, 2020 | Yes | Yes | Yes | Yes | Yes | No | Yes | Yes | Yes | Yes |
| **Hazra *et al.***, 2018 | Yes | Yes | Yes | Yes | No | No | No | Yes | Yes | Yes |
| **Huda *et al.***, 2018 | Yes | Yes | Yes | Yes | Yes | No | Yes | Yes | Yes | Yes |
| **Ilozumba *et al.***, 2018 | Yes | Yes | Yes | Yes | Yes | No | Yes | Yes | Yes | Yes |
| **McBride *et al.***, 2018 | Yes | Yes | Yes | Yes | Yes | No | No | No | Yes | Yes |
| **Nyema-Mudenda *et al.***, 2017 | Yes | Yes | Yes | Yes | Yes | No | No | Yes | Yes | Yes |
| **Shelus *et al.***, 2017 | Yes | Yes | Yes | Yes | Yes | No | Yes | Yes | Yes | Yes |
| **Velloza *et al*.**, 2019 | Yes | Yes | Yes | Yes | Yes | No | Yes | Yes | Yes | Yes |

|  | **Table S3. Thematic Analysis** | | |
| --- | --- | --- | --- |
|  | Positively transformational influences | Negatively transformational influences | Non-transformative influences |
| **Alam *et al.***, 2020 | Yes | Yes |  |
| **Alam *et al.***, 2019 | Yes |  |  |
| **Atukunda *et al.***, 2017 | Yes | Yes |  |
| **Brinkel *et al.***, 2017 | Yes |  |  |
| **Brown *et al.***, 2019 | Yes | Yes | Yes |
| **Campbell *et al.***, 2017 | Yes |  |  |
| **Decker *et al.***, 2020 | Yes |  |  |
| **Hazra *et al.***, 2018 | Yes | Yes |  |
| **Huda *et al.***, 2018 | Yes |  | Yes |
| **Ilozumba *et al.***, 2018 | Yes | Yes |  |
| **McBride *et al.***, 2018 | Yes |  |  |
| **Nyema-Mudenda *et al.***, 2017 | Yes | Yes |  |
| **Shelus *et al.***, 2017 | Yes |  |  |
| **Velloza *et al*.**, 2019 | Yes | Yes |  |

| **Table S4. End-user involvement** | |
| --- | --- |
|  | **Was the intervention co-designed with users / local communities?** |
| **Alam *et al.***, 2020 | Yes, extensive formative process outlined by authors |
| **Alam *et al.***, 2019 | Yes, extensive formative research prior to pilot |
| **Atukunda *et al.***, 2017 | No |
| **Brinkel *et al.***, 2017 | No |
| **Brown *et al.***, 2019 | No |
| **Campbell *et al.***, 2017 | No, but designed based on a prior acceptability survey |
| **Decker *et al.***, 2020 | Yes, community-participatory formative process used to adapt the myPlan app to fit local context prior to piloting / rollout |
| **Hazra *et al.***, 2018 | No |
| **Huda *et al.***, 2018 | No |
| **Ilozumba *et al.***, 2018 | No |
| **McBride *et al.***, 2018 | No, but held FGDs and interviews with CHWs and women regarding what was ‘lacking’ with prior interventions / methods of communication to inform mHealth intervention |
| **Nyema-Mudenda *et al.***, 2017 | No |
| **Shelus *et al.***, 2017 | No |
| **Velloza *et al*.**, 2019 | No, but reviewed the prototype with mHealth users prior and adapted accordingly |

| **Table S5. Data collection methods** | | |
| --- | --- | --- |
|  | **What qualitative methods were used?** | **Who was involved? *** |
| **Alam *et al.***, 2020 | In-depth interviews | In-depth interview with women only. |
| **Alam *et al.***, 2019 | In-depth interviews | In-depth interviews conducted with both women subscribers and their husbands. |
| **Atukunda *et al.***, 2017 | In-depth interviews | In-depth interviews conducted with ‘social supporters’ of the mHealth user (i.e., men and women) |
| **Brinkel *et al.***, 2017 | Focus group discussions | Focus group discussions held with mothers only. |
| **Brown *et al.***, 2019 | In-depth interviews | Interviews held with women only. |
| **Campbell *et al.***, 2017 | In-depth interviews | Interviews held with both men and women. |
| **Decker *et al.***, 2020 | In-depth interviews | Interviews held with women only. |
| **Hazra *et al.***, 2018 | In-depth interviews | In-depth interviews held with both women and their husbands. |
| **Huda *et al.***, 2018 | In-depth interviews; FGDs | In-depth interviews held with women and their husbands. FGDs held with mothers-in-law. |
| **Ilozumba *et al.***, 2018 | In-depth interviews | Interviews held with both men and women. |
| **McBride *et al.***, 2018 | In-depth interviews; FGDs | Interviews and FGDs held with women only. |
| **Nyema-Mudenda *et al.***, 2017 | In-depth interviews; FGDs | Interview and FGDs held with women only. |
| **Shelus *et al.***, 2017 | In-depth interviews | Interviews held with women only. |
| **Velloza *et al*.**, 2019 | In-depth interviews | Interviews held with both men and women. |

* This does not include any data collected from healthcare workers, etc – only women +/- their male partners.
